# Editorial: Model Organisms: A Precious Resource for the Understanding of Molecular Mechanisms Underlying Human Physiology and Disease

**DOI:** 10.3389/fgene.2019.00822

**Published:** 2019-09-10

**Authors:** Maria Grazia Giansanti, Roberta Fraschini

**Affiliations:** ^1^Istituto di Biologia e Patologia Molecolari del CNR, Dipartimento di Biologia e Biotecnologie, Università Sapienza di Roma, Roma, Italy; ^2^Dipartimento di Biotecnologie e Bioscienze, Università degli Studi di Milano-Bicocca, Milano, Italy

**Keywords:** model organisms, human diseases, human physiological model, DNA damage, human disorders

## Introduction

This issue includes eight reviews and five research articles, which highlight how research in model organisms lays the foundations for the comprehension of molecular mechanisms underlying human diseases. Although budding yeast and humans are separated by a billion years of evolutionary history, more than 400 essential yeast genes can be replaced with their human orthologs. Pioneering genetic studies in yeast have contributed to understand the mechanisms involved in autophagy and vesicle trafficking, two processes involved in cancer and neurodegenerative disorders (Novick et al., 1980; Takeshige et al., 1992; Mizushima et al., 1998). More recently, production of yeast strains expressing human genes (“humanized yeast”) has been essential for the detailed analysis of normal and pathogenic variants (Laurent et al., 2016). *Drosophila melanogaster* provides an extremely valid resource to investigate the mechanisms involved in organ formation and in the pathology of human diseases. Nearly 65% of human genes have orthologs in *D. melanogaster*, and nearly 75% of the genes involved in human disease have functional orthologs in flies (Reiter et al., 2001; Chien et al., 2002). The sophisticated genetic tools offered by *Drosophila* allow rapid generation of models for human disease, assaying the functional effects of human variant alleles and testing new therapeutic drugs (Moulton and Letsou, 2016; Wangler et al., 2017). *Danio rerio* shares vertebrate-conserved characteristics with human including very similar organs and is a highly suitable model system for investigating gene functions involved in hematopoiesis and screening for novel potential drugs (Wangler et al., 2017). Mouse models of human diseases are the most commonly used, reflecting the genetic and physiological similarities between humans and mice (Perlman, 2016).

### Using Budding Yeast to Study the Molecular Pathways That Are Altered in Human Diseases

Orlandi et al. used yeast as a model system to study aging of post-mitotic mammalian cells. Their data describe a connection between nicotinamide adenine dinucleotide (NAD^+^) content, mitochondrial functionality, and chronological life span. They show that, during chronological aging, an altered expression of the specific mitochondrial NAD^+^ carriers deeply influences the metabolic reprogramming that enables cells to acquire features required to maintain viability during aging.

Ohkuni et al. used yeast as a cellular model of neurodegenerative disorders such as Huntington’s disease (HD). They show that the SUMO-targeted ubiquitin ligase (STUbL) Slx5 reduces the toxicity and abnormal transcriptional activity associated with a mutant fragment of huntingtin (Htt) that induces aggregation, the causative agent of HD. Importantly, RNF4, the human ortholog of Slx5, limits the aberrant transcriptional activity of aggregation-prone Htt in yeast and in several cultured human cell lines. Thus, this study uncovers a conserved pathway that counteracts the accumulation of aggregating, transcriptionally active Htt, on chromatin in both yeast and in mammalian cells.

Fraschini’s review is focused on the molecular pathways and proteins involved in the control mitotic spindle morphogenesis and function, which are highly conserved from yeast to humans and whose impairment is connected with the development of human diseases. Fraschini illustrates the processes of spindle formation and orientation in yeast and in humans and includes many examples of misregulation that lead to the development of cancer and other human diseases.

Smurova and De Wulf report the role of centromeres and kinetochores in preserving genetic stability; in particular, they describe how centromere transcription contributes to faithful kinetochore function, how pericentromeric chromatin is silenced by RNA processing, and the transcriptional misregulation of (peri)centromeres during stress, natural aging, and disease.

Bonetti et al. illustrate how DNA ends are processed in budding yeast in order to maintain genome stability. DNA double-strand breaks (DSBs) are dangerous lesions that can be repaired by homologous recombination (HR) that occurs after DNA ends are correctly processed by several nucleases by a process called DNA end resection. The same nucleases function also during DNA replication in the processing of replication fork structures. The authors describe current knowledge of the mechanism of resection at DNA DSBs and replication forks.

Natali and Rancati describe the mutator phenotype in budding yeast. The mutator phenotype enhances genome instability and generation of phenotypic variation in a population of cells and increases the probability that some of these variations undergo selection and clonal expansion in challenging environments. This issue is particularly relevant for human health since cancer cells experience an increased mutational charge during early steps of carcinogenesis. The authors discuss the activity of the DNA replication and repair machineries and how mutations can confer increased genome instability. In addition, they describe recent clinical evidences in cancer biology indicating that these lessons can be applied to tumor development.

### Using Model Organisms to Model Tumor Formation and Progression

Sollazzo et al. used *Drosophila* larval wing epithelium to investigate the impact of MYC upregulation on cells carrying mutations in neoplastic tumor suppressor genes (nTSGs). MYC overexpression confers to cells mutant for different nTSGs, the ability to initiate multifocal, three-dimensional growth, a hallmark of mammalian pre-cancerous fields.

By using three different models of Ras induction and tumor formation in zebrafish, Anelli et al. demonstrate that six microRNAs increase following expression of a constitutively active *HRAS^G12V^* allele. Two Ras-induced microRNAs, namely, miR-146a and miR93a, target the Jmjd6 gene, which encodes a JumonjiC domain protein. Results in this study show that Jmjd6 plays a critical role in zebrafish melanoma development and that miR-146a and miR93a function as tumor suppressors, antagonizing Jmjd6 activation.

Mirzoyan et al. provide a comprehensive insight into the signaling pathways involved in tumorigenesis that are conserved in flies and highlight the ease to genetically manipulate these circuits to study cancer biology. In addition, they describe examples of *Drosophila* cancer models and their use to identify new therapeutic strategies.

Merigliano et al. describe the link between vitamin B6, diabetes, and cancer. Although several data indicate that diabetes and cancer are correlated, the molecular mechanisms involved remain to be clarified. Recent results obtained in *Drosophila* indicate that vitamin B6 deficiency causes hyperglycemia and increases DNA damage. These data suggest that, in diabetic patients, high PLP levels should increase the frequency of DNA damage thus contributing to cancer formation and progression.

### Using Model Organisms to Study the Neurological Defects Associated With Human Diseases

Burla et al. (2018) demonstrate that mutant mice with progeroid traits, caused by reduced expression of the *Ft1* gene (the ortholog of human *AKTIP*), display repeated seizures not linked to overt brain morphological alterations or severe neurodegeneration. However, Ft1 reduction is associated with the activation of the inflammatory markers IL-6 and TGF-β. Remarkably, reduction of the guardian of the genome p53 rescues the epileptic behavior and reverses back the expression of IL-6 and TGF-β in *Ft1* mutant mice, suggesting an involvement of DNA damage response in these phenotypes.

Two reviews describe the use of *D. melanogaster* for dissecting the molecular mechanisms underlying the neurological defects in inherited human diseases. Congenital disorders of glycosylation (CDGs) are multisystemic diseases caused by mutations in genes controlling the glycosylation pathways (Freeze et al., 2015). Most CDGs are associated with neurological defects, including mental retardation and seizures. As described by Frappaolo et al. *D. melanogaster* is emerging as a well-suited model organism for modeling congenital disorders of N-linked glycoprotein glycosylation due to a well-characterized glycome and a plethora of electrophysiological and behavioral assays that can be used to test neurological alterations in the whole organism.

The fragile-X (Fra-X) syndrome, caused by mutations in the fragile-X mental retardation (*Fmr1*) is associated with intellectual disability, autism, hyperactivity and language delay, long face and large ears, macroorchidism, and irregular spermatids (Santoro et al., 2012). The *Drosophila* Fra-X disease model recapitulates many phenotypic aspects of the Fra-X syndrome including defective neuronal architecture and synaptic function and altered germline development. Specchia et al. describe the involvement of dFmr1/FMRP protein in the piRNA pathway and in the DNA genome response, which may open up new perspectives in the search of potential therapeutic targets.

## Author Contributions

MGG and RF wrote the editorial.

## Funding

RF researches are supported by grants from PRIN (Progetti di Ricerca di Interesse Nazionale) and from the University of Milano Bicocca (FA). Financial support from the Italian Ministry of University and Research (MIUR) through grant “Dipartimenti di Eccellenza- 2017 “to University of Milano Bicocca, Department of Biotechnology and Biosciences is also acknowledged. Research in MG lab is supported by a grant from Fondazione AIRC per la ricerca sul Cancro (AIRC), grant IG 2017, Id 20779.

## Conflict of Interest Statement

The authors declare that the research was conducted in the absence of any commercial or financial relationships that could be construed as a potential conflict of interest.
